# Association of work‐time control with sickness absence due to musculoskeletal and mental disorders: An occupational cohort study

**DOI:** 10.1002/1348-9585.12181

**Published:** 2020-12-13

**Authors:** Sophie Charlotte Albrecht, Constanze Leineweber, Anneli Ojajärvi, Tuula Oksanen, Goran Kecklund, Mikko Härmä

**Affiliations:** ^1^ Stress Research Institute Department of Psychology Stockholm University Stockholm Sweden; ^2^ Finnish Institute of Occupational Health Helsinki Finland; ^3^ Institute of Public Health and Clinical Nutrition University of Eastern Finland Kuopio Finland

**Keywords:** autonomy, cohort study, depressive symptoms, flexible working hours, prospective study, psychosocial work environment

## Abstract

**Objectives:**

Work‐time control is associated with lower sickness absence rates, but it remains unclear whether this association differs by type of diagnosis and sub‐dimension of work‐time control (control over daily hours and control over time off) and whether certain vulnerable groups benefit more from higher levels of work‐time control.

**Methods:**

Survey data from the Finnish 10‐town study in 2004 were used to examine if baseline levels of work‐time control were associated with register data on diagnose‐specific sickness absence for 7 consecutive years (n = 22 599). Cox proportional hazard models were conducted, adjusted for age, sex, education, occupational status, shift work including nights, and physical/mental workload.

**Results:**

During follow‐up, 2,818 individuals were on sick leave (≥10 days) due to musculoskeletal disorders and 1724 due to mental disorders. Employees with high (HR = 0.80, 95% CI 0.74‐0.87; HR = 0.76, 95% CI 0.70‐0.82, respectively) and moderate (HR = 0.83, 95% CI 0.77‐0.90; HR = 0.85, 95% CI 0.79‐0.91, respectively) levels of control over daily hours/control over time off had a decreased risk of sickness absence due to musculoskeletal disorders. Sub‐group analyses revealed that especially workers who were older benefitted the most from higher levels of work‐time control. Neither sub‐dimension of work‐time control was related to sickness absence due to mental disorders.

**Conclusions:**

Over a 7‐year period of follow‐up, high and moderate levels of work‐time control were related to lower rates of sickness absence due to musculoskeletal disorders, but not due to mental disorders.

## 1 INTRODUCTION

Sickness absence (SA) is a major health concern—not only is SA associated with considerable distress for the individual in terms of decreased well‐being, income losses, and social isolation, but it also increases health‐care costs while decreasing tax incomes on the societal level. Sweden, for instance, lost approximately 57 billion SEK due to SA in 2015.^1^

SA and health status are strongly related but not identical. While short‐term SA may occur for a variety of reasons ranging from ill‐health to using sick leave as a form of coping mechanism, long‐term SA is a sound predictor of both morbidity and mortality.^2^ Long periods of decreased health and well‐being often precede long‐term SA.^3^, ^4^ If underlying health problems increase the risk for future SA, keeping an individual in work may be more likely if their workload can be adapted to current needs. One way to achieve this could be to increase an individual's influence on work demands and decision making.^5^ Another way to cope with demands stemming from inside and outside of work could be to utilize control over working time, for instance when to take time off from work or adapt timing of work, here defined as “an individual's autonomy regarding duration and distribution of working time”.^6^

Work‐time control (WTC) has been found to be associated with a number of health outcomes. For example, low levels of WTC were linked to more depressive symptoms,^7^ higher psychological distress,^8^ and more sleep disturbances.^9^ Moreover, disability pension due to musculoskeletal disorders and mental disorders was associated with lower perceived levels of WTC.^10^ These studies indicate that higher levels of WTC may be helpful in critical times to manage workload and potentially prevent subsequent long‐term SA. Still, only a few studies have investigated whether low levels of WTC increase risk for SA or if high levels of WTC can buffer against SA. For instance, Ala‐Mursula et al (2002) found that, for women, low WTC increased risk of SA of more than 3 days. A Swedish study concluded that increasing WTC for those with low levels of control could result in a reduction up to 12% in SA (more than 30 days), depending on the type of occupation.^11^

Differential effects of the two sub‐dimensions of WTC on health outcomes are rarely investigated.^12^ In a previous study, we found control over time off to be more strongly associated with depressive symptoms than control over daily hours.^7^ Another study showed that while control over time off had a direct effect on work‐life interference (which is related to a number of health outcomes), control over daily hours only moderated effects from long working hours to work‐life interference.^13^ Likewise, we recently found that effects from WTC on work‐life interference and depressive and musculoskeletal symptoms were stronger for control over time off compared to control over daily hours.^14^

Few studies have looked upon causal mechanisms underlying effects of WTC on health. High WTC has been related to lower levels of work‐family conflict and emotional exhaustion^12^ which in turn could buffer against SA due to mental disorders such as depression, anxiety, or stress‐related disorders.^15^ Furthermore, WTC may be a useful tool to prevent developing physical health issues. The opportunity to take breaks or days off and avoid working long hours before or as soon as for instance musculoskeletal symptoms first appear may prevent psychological strain and decrease biomechanical load which in turn should decrease risk for musculoskeletal disorders.^16^ Mental and musculoskeletal disorders are major causes for SA.^17^ To the best of our knowledge, effects of WTC on diagnose‐specific SA have not been examined to date.

The present study aimed to investigate whether higher levels of WTC are associated with a decreased risk for SA due to musculoskeletal or mental disorders. As previous research found two sub‐dimensions underlying the concept of WTC,^18^, ^19^, ^20^ but previous research on sickness absence used only general indicators of WTC, we differentiate between control over daily hours and control over time off. Furthermore, we were interested if certain vulnerable groups that commonly show high levels of SA might benefit more from high WTC. To this end, we investigated if effects of WTC on SA are modified by sex,^8^ age,^21^ education,^22^ occupational class,^21^ physical and mental workload,^22^ and musculoskeletal or mental health.^21^, ^23^ We expect that those who are more prone to SA, that is, women, those with older age, lower education, lower occupational class, higher workload, and poor health, will gain more by high work‐time control than those who are less prone to SA.

## 2 METHODS

**FIGURE 1:**
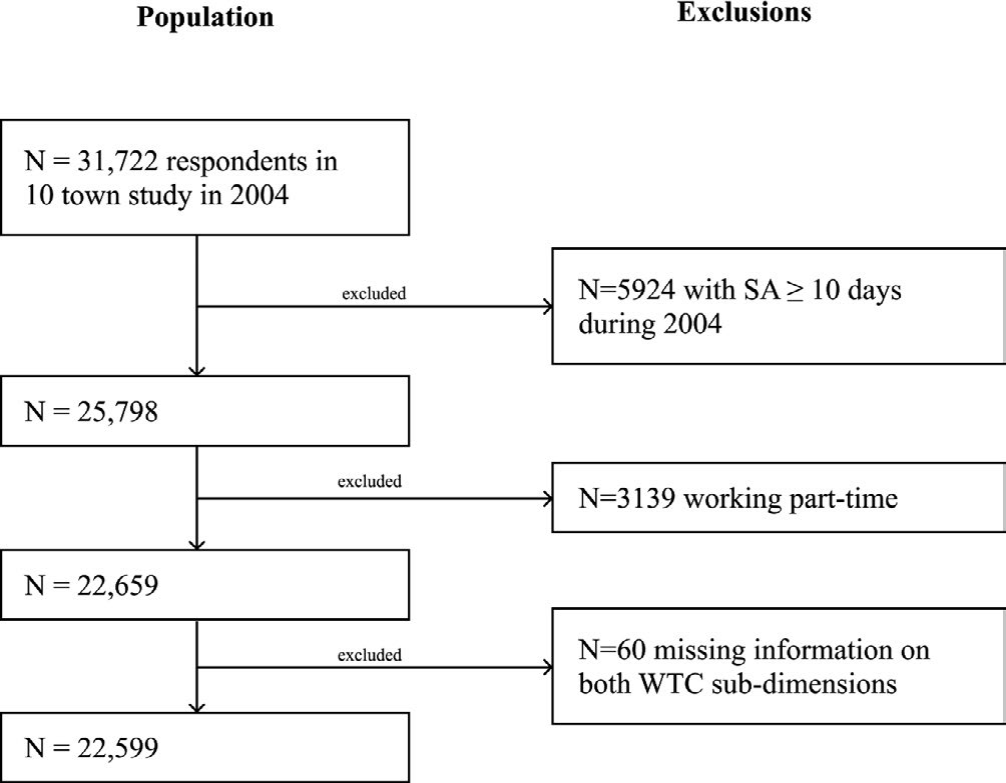
Sample selection procedure

Participants were drawn from the Finnish Public Sector Study, which is a prospective occupational cohort study with identifiable questionnaire surveys. The current sample is based on participants from the 10 towns only, working in a wide range of occupations (from administrative personnel and professionals to semi‐skilled and unskilled workers). In 2004, altogether 31 722 employees in the service of 10 municipalities responded to the survey (response rate 65%). After exclusion of those with any pension or SA of ≥10 days during 2004 (n = 5924), those working part‐time (n = 3139), and those missing information on both WTC subscales (n = 60), the final sample consisted of 22 599 employees (Figure 1). The Finnish Public Sector Study has been approved by the Ethics Committee of the Helsinki Uusimaa Hospital district (HUS 1210/2016).

### 2.1 Sickness absence

Diagnose‐specific data on SA between 2005 and 2011 was obtained from national sickness insurance register, maintained by the Social Insurance Institution of Finland, where all sick leaves exceeding 9 days are reliably recorded regarding the respective main diagnosis. Here, we considered only days of full‐time sickness absence. Musculoskeletal disorders covered ICD‐10 codes M05‐M13, M30‐M36, M50‐M54, and M79.7. Mental disorders covered both affective (F32‐34) and anxiety (F40‐F42) disorders.

### 2.2 Work‐time control

WTC was measured using a 7‐item index assessing employees' perceived control over length of a work day, starting and ending times, taking breaks, running private errands during work, which days to work, scheduling of vacation and unpaid leave.^8^ Items are rated on a 5‐point Likert scale regarding the level of control from 1 (=very little) to 5 (=very much). A principal component analysis with varimax rotation (eigenvalue criterion >1) confirmed two underlying sub‐dimensions. “Control over daily hours” (56.29% of the total variance, Cronbach's α = 0.86, mean = 3.71, SD = 1.17) contained the items on length of a work day and starting and ending times. “Control over time off” (25.32% of the total variance, Cronbach's α = 0.66, mean = 2.81, SD = 1.00) covered two items on scheduling vacation and unpaid leave. The remaining three items (taking breaks, running private errands during work, which days to work) were excluded from further analyses due to high double loadings. For each sub‐dimension, means were calculated for each individual and the scale was split into tertiles to reflect low, moderate, and high levels of control.

### 2.3 Covariates and moderators

Sex, age, and occupational status were derived from employers' registers. Educational attainment, shift work, physical, and mental workload, and self‐rated symptoms of mental disorders were obtained from questionnaire data. All were assessed as potential confounders and/or moderators. Sex was measured binary (men vs women), age was divided into three age groups (≤39, 40‐49, ≥50). Education was measured in terms of highest attained education (primary, lower secondary, upper secondary school, university or similar) and occupational status was dichotomized into blue‐ vs white‐collar workers. Shift work was measured by a single question: “Do you regularly work daytime hours?” (“yes”/“no”). Physical and mental workload was measured each by a single question: “How heavy or light do you think your work is?” Response options for physical workload ranged from 1=“very light” to 4=“very heavy” (which was further dichotomized into low and high physical workload) and for mental workload from 1=“very or quite light” to 3=“very heavy.” Self‐reported musculoskeletal symptoms and symptoms of mental disorders were measured by a single question: “Has a doctor told you that you have or have had …” followed by a number of different complaints and diseases, answered with “yes” or “no.” Those who answered “yes” to at least one item of musculoskeletal (wear of the joints or osteoarthritis, rheumatoid arthritis, soft tissue rheumatism, sciatica) or mental disorder (depression, other mental disorder), respectively, were coded as suffering from musculoskeletal or mental disorder, respectively. Furthermore, used self‐reported levels of daily working hours (<7.5, 7.5‐8.0, >8.0 hours), daily overtime hours (no overtime, <1 hour, >1 hour), and hours of sleep (≤6.5, 7.0‐8.5, ≥9 hours) to describe our selected sample.

### 2.4 Statistical analysis

Data were analyzed separately for SA due to musculoskeletal disorders and due to mental disorders. Associations of control over daily hours and time off with SA rates were calculated using Cox Proportional‐Hazards Models. Hazard ratios (HR) are presented with 95% confidence intervals (CI). The follow‐up period started the year after the survey (January 1, 2005) and ended with first incidence of SA of ≥10 days, retirement, disability retirement, emigration, death or December 31, 2011—whichever came first.

In the first step, crude associations between the two sub‐dimensions of WTC and SA (≥10 days) were calculated. Secondly, we controlled for age, sex, education, occupational status, shift work including nights, and physical workload. Next, we investigated which variables played a moderating role in the relationship between WTC and SA. Interaction terms with WTC were separately included in the models for sex, age, education, occupational status, physical and mental workload, and baseline self‐rated mental and musculoskeletal health (while controlling for other covariates). Group‐based models were estimated to describe differences in HRs between the levels of moderating variables. These analyses were controlled for the family‐wise error rate using the Bonferroni correction resulting in a significance level of *P* < .006. All analyses were conducted with SAS, version 9.4, statistical software (SAS Institute, Inc) using the PHREG procedure.

## 3 RESULTS

### 3.1 Baseline descriptives

Of the participants (n = 22 599), 75% were women, 39% were 50 years old or older and 83% were non‐manual workers. Temporary work contracts were rare and over 60% of participants experienced either light or quite light mental and physical workload. Poor mental health was reported by 13% of participants while 31% reported poor physical health at baseline. Table 1 shows descriptive statistics at baseline of the study population differentiating between control over daily hours and control over time off. High control over daily hours was found more often among those who were women, older, better educated, white‐collar workers, working daytime hours, working on temporary contracts, with low physical and mental workload, and with better physical health at baseline. Similar results were found for control over time off, but a few differences could be noted: permanent workers reported more often high control over time off as compared to those with temporary work contracts; while those with poor baseline mental health reported more often low control over time off, this was not the case regarding control over daily hours.

**TABLE 1:** Descriptive frequencies of the study sample at baseline differentiating between levels of control over daily hours and control over time off

	Total n (%)	Control over daily hours				Control over time off			
		High	Moderate	Low	*P*‐value	High	Moderate	Low	*P*‐value
		n (%)	n (%)	n (%)		n (%)	n (%)	n (%)	
All	22 599	8071	7683	6693		7694	7927	6595	
Sex					*P* < .001				*P* = .016
Men	5632 (25)	2176 (39)	1462 (25)	1965 (35)		1998 (35)	1902 (34)	1634 (29)	
Women	16 967 (75)	5895 (35)	6221 (37)	4728 (29)		5696 (34)	6025 (36)	4961 (29)	
Age					*P* < .001				*P* < .001
≤39	6029 (27)	2091 (35)	2252 (37)	1655 (27)		2095 (35)	2085 (34)	1763 (29)	
40‐49	7870 (35)	2934 (37)	2643 (34)	2239 (28)		2811 (36)	2803 (36)	2137 (27)	
≥50	8700 (38)	3046 (35)	2788 (32)	2799 (32)		2788 (32)	3039 (35)	2695 (31)	
Education					*P* < .001				*P* < .001
Primary	4402 (20)	928 (21)	1140 (33)	1989 (46)		1134 (27)	1733 (40)	1430 (33)	
Lower secondary	6537 (29)	2061 (32)	2175 (34)	2255 (35)		2267 (35)	2590 (40)	1559 (24)	
Upper secondary	5718 (26)	2244 (39)	2021 (36)	1427 (25)		2350 (42)	2235 (40)	1064 (19)	
University or similar	5567 (25)	2724 (49)	1928 (35)	886 (16)		1831 (33)	1226 (22)	2434 (44)	
Occupational class					*P* < .001				
Blue collar	3662 (16)	557 (15)	1148 (31)	1911 (52)		986 (26)	1335 (36)	1244 (34)	
White collar	18 937 (84)	7514 (40)	6535 (34)	4792 (25)		6708 (35)	6592 (35)	5351 (28)	
Type of shift work					*P* < .001				*P* < .001
Daytime only	19 909 (88)	7665 (38)	6823 (34)	5287 (26)		7048 (35)	6842 (36)	5689 (29)	
Including nights	2056 (9)	258 (12)	677 (33)	1113 (54)		461 (22)	839 (41)	721 (35)	
Type of work contract					*P* < .001				*P* < .001
Permanent	20 563 (91)	7283 (35)	6344 (31)	6197 (30)		7104 (34)	7325 (21)	5792 (28)	
Temporary	1998 (8)	764 (38)	730 (36)	491 (24)		569 (28)	588 (29)	801 (40)	
Physical workload					*P* < .001				*P* < .001
Low	14 070 (63)	6618 (47)	4554 (32)	2817 (20)		5514 (39)	4590 (33)	3738 (27)	
High	8335 (37)	1382 (17)	3676 (37)	3815 (46)		2119 (25)	3269 (39)	2799 (34)	
Mental workload					*P* < .001				*P* < .001
Very or quite light	4552 (20)	1703 (37)	1401 (31)	1423 (31)		1815 (40)	1693 (37)	956 (21)	
Quite heavy	13 510 (60)	4853 (36)	4814 (36)	1379 (28)		4583 (34)	4846 (36)	3871 (29)	
Very heavy	4270 (19)	1423 (33)	3761 (32)	1436 (34)		1228 (29)	1284 (30)	1694 (40)	
Mental health at baseline					*P* = .628				*P* = .007
Good	18 469 (82)	6620 (36)	6298 (34)	5443 (29)		6411 (34)	6463 (35)	4307 (23)	
Poor	2712 (12)	994 (37)	922 (34)	778 (29)		881 (32)	934 (34)	855 (32)	
Physical health at baseline					*P* < .001				*P* < .001
Good	14 948 (66)	5532 (37)	5118 (34)	4216 (28)		5269 (35)	5223 (35)	4218 (28)	
Poor	6592 (29)	2163 (33)	2217 (34)	2159 (33)		2117 (32)	2301 (35)	2070 (31)	
Sleep hours					*P* < .001				*P* = .0113
≤6.5 h	4948 (22)	1694 (35)	1602 (33)	1616 (33)		1580 (33)	1772 (37)	1496 (31)	
7.0‐8.5 h	16 760 (75)	6105 (37)	5777 (35)	4775 (29)		5829 (35)	5846 (35)	4822 (30)	
≥9.0 h	615 (3)	194 (32)	199 (33)	215 (35)		213 (35)	210 (35)	181 (30)	
Daily working hours					*P* < .001				*P* < .001
<7.5 h	5898 (26)	3141 (54)	1862 (32)	864 (15)		2072 (36)	1661 (29)	2067 (36)	
7.5‐8.0 h	15 023 (67)	4642 (31)	5380 (36)	4895 (33)		5148 (35)	5644 (38)	3975 (27)	
>8.0 h	1678 (7)	288 (17)	441 (27)	934 (56)		474 (29)	622 (38)	553 (34)	
Overtime hours per day					*P* < .001				*P* < .001
No overtime	5531 (25)	1779 (32)	1811 (33)	1941 (35)		2046 (37)	2065 (38)	1379 (25)	
≤1 h	5785 (26)	2720 (47)	1968 (34)	1097 (19)		2194 (38)	1960 (34)	1607 (28)	
>1 h	2942 (13)	1217 (41)	988 (34)	737 (25)		782 (27)	748 (25)	1402 (48)	
	8189 (36)	2355 (29)	2916 (36)	2918 (36)		2672 (33)	3154 (39)	2207 (28)	

### 3.2 Sickness absence due to musculoskeletal disorders

During follow‐up, 2,818 individuals were on sick leave (≥10 days) due to musculoskeletal disorders and 1724 due to mental disorders. Table 2 shows the associations between levels of WTC and risk of SA. Crude estimates decreased slightly but remained significant after adjusting for potential confounders. The risk of sick leave due to musculoskeletal disorders decreased for those with moderate (HR = 0.83, 95% CI = 0.77 to 0.90) and high (HR = 0.80, 95% CI = 0.74 to 0.87) levels of control over daily hours compared to those with low levels of control. Likewise, associations were significant for control over time off regarding moderate (HR = 0.85, 95% CI = 0.79 to 0.91) and high levels of control (HR = 0.76, 95% CI = 0.70 to 0.82).

**TABLE 2:** Hazard ratios (95% CI) for sickness absence (≥10 d) due to musculoskeletal and mental disorder by levels of work‐time control at baseline (low levels of control as reference)

Level of control	Sickness absence due to musculoskeletal disorders		Sickness absence due to mental disorders	
	Unadjusted	Adjusted^a^	Unadjusted	Adjusted^a^
Control over daily hours				
Moderate	0.66 (0.61‐0.71)***	0.83 (0.77‐0.90)***	1.03 (0.93‐1.14)	1.08 (0.97‐1.21)
High	**0.50 (0.46‐0.54)** ***	**0.80 (0.74‐0.87)** ***	0.93 (0.84‐1.03)	1.06 (0.95‐1.19)
Control over time off				
Moderate	**0.90 (0.83‐0.96)** **	**0.85 (0.79‐0.91)** ***	1.05 (0.94‐1.16)	1.05 (0.95‐1.17)
High	**0.67 (0.62‐0.73)** ***	**0.76 (0.70‐0.82)** ***	0.92 (0.83‐1.01)	0.95 (0.86‐1.06)

^a^ adjusted for age, sex, education, occupational class, shift work including nights, and physical workload

*** p<.001;**p<.01

Next, we examined whether certain groups benefit more from higher levels of WTC. Potential moderating effects were investigated regarding the relationship between control over daily hours/time off and SA (Table 3). We found statistically significant interactions between control over daily hours and age (*P* = .002) and occupational class (*P* = .005). Specifically, higher levels of control over daily hours were more protective against SA due to musculoskeletal disorders among older (≥40 years old) and white‐collar employees. Control over time off significantly interacted only with age (*P* = .001); those who were older benefited most from higher levels of control over time off.

**TABLE 3:** Moderation analyses: Hazard ratios (95% CI) for sickness absence (≥10 d) due to musculoskeletal disorder by levels of control over daily hours and control over time off at baseline (adjusted results^a^; significance level after Bonferroni correction at 0.006)

Moderator variable	*P*‐value interaction term	Control over daily hours		*P*‐value interaction term	Control over time off	
		Moderate vs low	High vs low		Moderate vs low	High vs low
		HR (95% CI)	HR (95% CI)		HR (95% CI)	HR (95% CI)
Sex	.984			.345		
Women		0.82 (0.76‐0.90)***	0.79 (0.72‐0.87)***		0.82 (0.75‐0.89)***	0.75 (0.68‐0.82)***
Men		0.85 (0.72‐0.99)*	0.86 (0.72‐1.03)		0.96 (0.82‐1.12)	0.82 (0.69‐0.97)*
Age	**.002**			**.001**		
Up to 39 y		1.04 (0.87‐1.23)	0.94 (0.77‐1.14)		1.02 (0.86‐1.22)	**0.81 (0.67‐0.98)**
40‐49 y		0.85 (0.76‐0.96)**	0.81 (0.70‐0.93)**		0.78 (0.70‐0.88)***	0.65 (0.57‐0.74)***
50 y and above		0.73 (0.65‐0.82)***	0.75 (0.65‐0.85)***		0.83 (0.74‐0.93)**	0.86 (0.76‐0.98)*
Education	.014			.011		
Elementary school		0.75 (0.66‐0.86)***	0.86 (0.74‐1.00)		0.81 (0.71‐0.92)**	0.81 (0.70‐0.94)**
Middle/primary school		0.90 (0.80‐1.02)	0.83 (0.72‐0.96)*		0.85 (0.75‐0.95)**	0.66 (0.57‐0.75)***
Secondary school		0.86 (0.72‐1.03)	0.66 (0.55‐0.81)***		0.79 (0.66‐0.96)*	0.77 (0.63‐0.93)**
University or similar		0.75 (0.57‐1.00)	**0.85 (0.65‐1.11)**		0.75 (0.57‐1.00)	0.85 (0.65‐1.11)
Occupational class	**.005**			.255		
White collar		0.81 (0.74‐0.88)***	0.75 (0.68‐0.83)***		0.88 (0.81‐0.97)**	0.80 (0.73‐0.88)***
Blue collar		0.85 (0.75‐0.98)*	1.02 (0.86‐1.21)		0.78 (0.68‐0.89)***	0.69 (0.59‐0.81)***
Physical workload	.031			.321		
Low		0.78 (0.69‐0.89)***	0.76 (0.67‐0.85)***		0.91 (0.80‐1.03)	0.85 (0.75‐0.96)**
High		0.86 (0.78‐0.94)**	0.88 (0.78‐0.997)*		0.82 (0.75‐0.91)***	0.72 (0.64‐0.81)***
Self‐reported musculoskeletal disorders	.008			.844		
No		0.92 (0.82‐1.03)	0.77 (0.67‐0.88)***		0.84 (0.74‐0.94)**	0.77 (0.68‐0.87)***
Yes		0.80 (0.72‐0.89)***	0.84 (0.74‐0.94)**		0.90 (0.81‐0.99)*	0.79 (0.70‐0.88)***
Mental workload	.081			.682		
Very or quite light		0.78 (0.66‐0.93)**	0.88 (0.73‐1.06)		0.93 (0.78‐1.11)	0.86 (0.72‐1.04)
Quite heavy		0.88 (0.80‐0.97)**	0.78 (0.69‐0.87)***		0.85 (0.77‐0.94)**	0.77 (0.69‐0.87)***
Very heavy		0.74 (0.63‐0.88)***	0.80 (0.63‐0.88)***		0.86 (0.73‐1.01)	0.73 (0.61‐0.87)***
Self‐reported mental disorders	.342			.991		
No		0.88 (0.81‐0.96)**	0.83 (0.76‐0.92)***		0.86 (0.79‐0.94)***	0.78 (0.71‐0.85)***
Yes		0.72 (0.60‐0.88)***	0.72 (0.58‐0.88)***		0.86 (0.71‐1.04)	0.76 (0.62‐0.93)**

^a^ Adjusted for age, sex, education, occupational status, shift work including nights, and physical workload; ***p<.001; **p<.01; *p<.05.

### 3.3 Sickness absence due to mental disorders

During follow‐up, 1724 individuals were on sick leave (≥10 days) due to mental disorders. Neither control over daily hours nor control over time off showed significant associations with SA due to mental disorders (Table 2). When exploring effect modification (Table 4), we found no significant interactions regarding both control over daily hours and control over time off.

**TABLE 4:** Moderation analyses: Hazard ratios (95% CI) for sickness absence (≥10 d) due to mental disorder by levels of control over daily hours and control over time off at baseline (adjusted results; significance level after Bonferroni correction at 0.006)

Moderator variable	Control over daily hours			Control over time off		
	*P*‐value interaction term	Moderate vs low	High vs low	*P*‐value interaction term	Moderate vs low	High vs low
		HR (95% CI)	HR (95% CI)		HR (95% CI)	HR (95% CI)
Sex	.189			.609		
Women		1.08 (0.96‐1.21)	1.11 (0.98‐1.25)		1.07 (0.96‐1.10)	0.97 (0.86‐1.10)
Men		1.08 (0.83‐1.42)	0.78 (0.59‐1.03)		0.97 (0.74‐1.26)	0.85 (0.65‐1.11)
Age	.538			.045		
Up to 39 y		1.04 (0.84‐1.28)	1.19 (0.96‐1.47)		1.21 (0.99‐1.49)	0.93 (0.75‐1.16)
40‐49 y		1.16 (0.98‐1.38)	1.07 (0.90‐1.28)		1.12 (0.94‐1.32)	0.98 (0.82‐1.17)
50 y and above		1.03 (0.86‐1.23)	0.96 (0.79‐1.16)		0.88 (0.74‐1.05)	0.93 (0.78‐1.12)
Education	.588			.724		
Elementary school		0.89 (0.70‐1.14)	0.97 (0.74‐1.27)		0.91 (0.71‐1.16)	0.81 (0.61‐1.07)
Middle/primary school		1.15 (0.97‐1.37)	1.05 (0.87‐1.27)		1.04 (0.87‐1.25)	0.95 (0.78‐1.15)
Secondary school		1.20 (0.98‐1.47)	1.13 (0.97‐1.40)		1.05 (0.85‐1.29)	0.98 (0.79‐1.22)
		1.00 (0.75‐1.33)	1.07 (0.81‐1.40)		1.26 (0.99‐1.60)	0.98 (0.78‐1.23)
Occupational class	.855			.713		
White collar		1.09 (0.97‐1.23)	1.06 (0.94‐1.20)		1.05 (0.93‐1.17)	0.94 (0.84‐1.06)
Blue collar		1.06 (0.81‐1.38)	1.12 (0.80‐1.55)		1.07 (0.81‐1.42)	1.01 (0.74‐1.38)
Physical workload	.045			.452		
Low		1.05 (0.90‐1.29)	1.11 (0.96‐1.29)		1.03 (0.90‐1.19)	0.90 (0.78‐1.03)
Highlow		1.14 (0.98‐1.32)	0.88 (0.72‐1.068		1.08 (0.92‐1.26)	1.01 (0.85‐1.21)
Self‐reported musculoskeletal disorders	.202			.011		
No		1.11 (0.97‐1.28)	1.04 (0.90‐1.20)		1.14 (0.93‐1.24)	1.07 (0.93‐1.24)
Yes		1.09 (0.90‐1.30)	1.19 (0.99‐1.43)		0.85 (0.71‐1.01)	0.78 (0.65‐0.94)**
Mental workload	.588			.588		
Very or quite light		0.92 (0.69‐1.23)	0.92 (0.69‐1.23)		1.13 (0.82‐1.56)	1.16 (0.84‐1.60)
Quite heavy		1.13 (0.97‐1.30)	1.19 (1.02‐1.37)		1.06 (0.92‐1.22)	0.91 (0.78‐1.05)
Very heavy		1.09 (0.90‐1.32)	0.93 (0.77‐1.14)		1.04 (0.87‐1.26)	0.98 (0.81‐1.18)
Self‐reported mental disorders	.142			.053		
No		1.02 (0.88‐1.18)	1.08 (0.94‐1.26)		1.12 (0.97‐1.31)	1.04 (0.90‐1.21)
Yes		1.26 (1.06‐1.49)	1.07 (0.90‐1.28)		0.89 (0.76‐1.06)	0.92 (0.78‐1.09)

## 4 DISCUSSION

In this large prospective cohort study, we found that with higher levels of WTC, the rate of SA decreased regarding musculoskeletal, but not mental disorders, during a follow‐up of 7 years. Individuals with high control over daily hours/time off had a 20%‐25% lower risk of SA due to musculoskeletal disorders (HR = 0.80/0.76). No differences were found between the two sub‐dimensions control over daily hours and control over time off. Moderation analyses revealed that especially workers who were older benefitted the most from higher levels of WTC in terms of reduced risk for SA due to musculoskeletal disorder.

To our knowledge, this is the first study to examine the association of WTC with diagnose‐specific SA due to mental and musculoskeletal disorders. The results are in line with previous research on general SA. One Finnish study showed that women with low levels of WTC had an increased risk for SA lasting at least 3 days.^8^ Moreover, WTC could diminish the negative effects of long working hours on SA in a subsequent study.^24^ Along the same line, a study found that employees with the highest WTC (upper quartile) had 0.6 times lower rates of SA (more than 10 days) as compared to those in the lowest WTC quartile.^21^ Still, none of these studies differentiated between particular diagnostical groups.

In our study, the rate of SA due to musculoskeletal disorders at any given point decreased by around 20% with moderate to high levels of control over daily hours and time off. This can be compared to findings by Vahtera et al (2010) who found a reduction around 50% in incidence of disability pension when high levels of WTC were reported. Unexpectedly, our study failed to find an association between WTC with subsequent SA due to mental disorders. These findings are in contrast to previous research that observed effects of WTC on self‐reported mental health outcomes such as depressive symptoms.^7^, ^25^ However, our study differs to others in that we utilized an “objective” measure of mental health with a high threshold to fall into that category (SA of 10 days or more due to mental diagnosis). As there is a high comorbidity of mental and musculoskeletal disorders,^26^ it is quite possible that doctors focus primarily on musculoskeletal disorders as reason for SA. As a result, evidence of effects of WTC is possibly clearer regarding subjectively rated mental health in contrast to more objective measures of health. Also, the number of cases with SA due to mental disorders was fairly low in our study, particularly for men. Thus, power issues might at least partly explain the results: confidence intervals were larger for results on mental disorders and indicate that with high levels of WTC, SA rates due to mental disorders may actually decrease to a small degree. A different explanation for our results lies in the fact that we examined full‐time SA only. While musculoskeletal disorders are the leading cause of SA in Finland, mental disorders are more commonly the reason for partial sick leave.^17^ Future studies should investigate rates of full and partial SA. Finally, the causal chains between psychosocial work factors—such as WTC—and musculoskeletal and mental disorders are still not understood well^16^ and future research might be able to shed light on diverging findings. Speculatively, high levels of WTC may not only prevent psychological stress but also decrease biomechanical load by allowing workers to take for instance short breaks and vacation when needed. As a result, WTC could help to avoid physical and psychological strain and thereby diminish risk of developing musculoskeletal problems.

In our results, we highlight that groups that are more prone to long‐term SA seem to benefit the most from higher levels of WTC. Particularly employees of 40 years and above were less at risk for SA due to musculoskeletal disorders with both moderate and high levels of control over daily hours and time off. This result is in line with what one would expect: with growing age, signs of wear in muscles and joints increase and thus the risk for developing musculoskeletal symptoms increases.^27^ Since older employees are more vulnerable to develop musculoskeletal problems, they may also benefit more from increased levels of WTC as a way of coping in and outside of work. Although the causal mechanisms are unclear, taking regular breaks and vacation when needed and avoiding long working hours may decrease both physical and psychological strain which in turn reduces the risk of musculoskeletal disorders.^16^ Younger employees might benefit from higher WTC on a more long‐term scale: WTC may diminish future risk of musculoskeletal disorders. With an average follow‐up time of 7 years, this study was unable to investigate this further.

No other interacting effects were found to be significant. Against our expectations, mental or physical health problems at baseline did not result in greater benefits from high levels of WTC. In contrast, a study examining the effect of WTC on extended working life found that irrespective of any somatic disease, all employees benefited from high WTC; but those with mental disorders benefited more than those without.^23^ Of the employees diagnosed with a mental disorder and reporting higher levels of WTC, 21% extended their working life by at least 6 months, while only 15% of those with low WTC and a mental disorder did so.

### 4.1 Strengths and limitations

A particular strength of this study is the use of reliable, register‐based outcome data on SA including specific diagnoses as well as a long follow‐up time. Participants were based on the Finnish Public Sector study—a large cohort study of public sector employees. Additionally, we were able to control for a number of potential confounding variables.

However, some limitations of this study need to be pointed out. Because of the study sample characteristics, results may not necessarily be generalizable to other populations such as more male‐dominated work sectors. We used baseline‐only measurements of WTC meaning the exposure to high or low WTC may not have been long enough to affect SA rates. Additionally, since we did not study long‐term trajectories of WTC after baseline, we do not know whether different trajectories of WTC could have differential effects on SA.

While we excluded participants with long‐term SA during the baseline year, we cannot fully exclude that prior rates of SA have influenced subjective ratings of WTC. Misclassification of diseases in favor of musculoskeletal disorders rather than mental disorders cannot be excluded and may be one reason for the insignificant findings regarding mental disorders. Although we included a number of potential confounders based on theoretical considerations, we cannot rule out bias due to unmeasured confounding in our results (eg, ethnicity, other factors of the work environment, genetical predisposition for musculoskeletal/mental disorders).

### 4.2 Concluding remarks

Higher WTC was related to decreased risk for SA due to musculoskeletal disorders, particularly for employees who were older. Increasing levels of WTC may be a useful tool to decrease long SA spells, particularly given that it is to some degree modifiable in most occupations. Future research needs to examine this, ideally in an intervention design. Employers interested in preventing and counteracting sickness absence to decrease costs and productivity losses may seek to increase employees' control over working hours, for instance by implementing flexible hours schedules (flexitime), increasing autonomy of scheduling vacation and promoting a culture at work of flexibly taking breaks whenever needed. These policies ideally should be anchored both in working contracts and the work place culture (with managers acting as role models). Particularly workers with reduced work ability (eg, due to advancing age) would benefit from increasing autonomy and self‐determination over working hours.^23^

## AUTHOR CONTRIBUTIONS

SCA, CL, and MH conceived the ideas; SCA, CL, and AO planned the data analysis; AO analyzed the data; SCA and CL led the writing of the manuscript with significant input on text, data, and interpretation of results from TO, GK, and MH

## DISCLOSURE

*Approval of the research protocol*: N/A. *Informed consent*: Participants of the Finnish Public Sector Study are informed about use of collected data and response to questionnaires acts as a form of written consent. *Registry and the registration no. of the study/trial*: HUS 1210/2016. *Animal studies*: N/A. *Conflict of interest*: The authors declare no conflict of interest for this article.
